# P-1143. Implementing Admission Screening for Candida auris and Carbapanemase-Producing Organisms (CPOs) in a Low-Prevalence State

**DOI:** 10.1093/ofid/ofaf695.1337

**Published:** 2026-01-11

**Authors:** Evelyn L A Donahoe, Adelina Mart, Heather Hertzel, Alexia Y Zhang, Dat Tran, Christopher D Pfeiffer

**Affiliations:** Oregon Health Authority, Portland, Oregon; Oregon Health Authority, Portland, Oregon; OR EIP, Portland, Oregon; Oregon Health Authority, Portland, Oregon; Oregon Health Authority, Portland, Oregon; VA Portland Health Care System, Portland, Oregon

## Abstract

**Background:**

In response to CDC guidance recommending admission screening for *Candida auris* and carbapenemase-producing organisms (CPOs), the Oregon Health Authority (OHA) launched a statewide effort to encourage and support healthcare facilities in implementing screening protocols. Oregon remains a low-prevalence state for these organisms.

**Figure 1:**
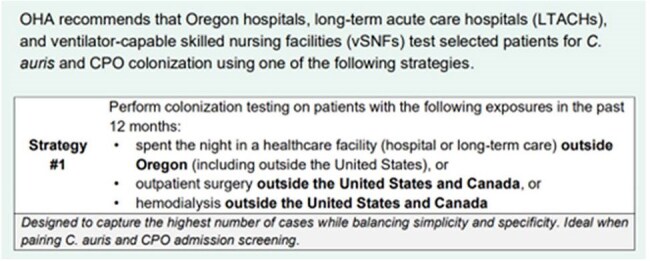
Screen shot from Oregon’s interim admission screening guidance Weblink: https://rebrand.ly/AdmissionScreening

**Figure 2:**
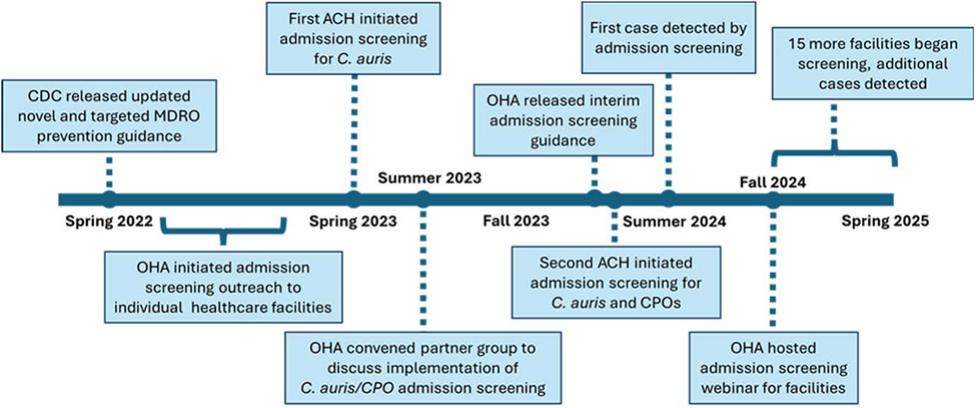
Timeline of C. auris and CPO admission screening implementation in Oregon

**Methods:**

Starting in 2022, OHA began outreach to all high acuity Oregon healthcare facilities: 63 acute care hospitals (ACHs), one long-term acute care hospital (LTACH), and one ventilator-capable skilled nursing facility (vSNF). Twenty-nine facilities were prioritized based on regional epidemiology and facility interest. Outreach included emails (14), response consultations (6), presentations (4), and a statewide webinar (1). Ten volunteer facilities helped develop Oregon-specific screening guidance, published April 2024 (figure 1). This guidance was shared during subsequent OHA-led infection prevention trainings and assessments.

**Figure 3:**
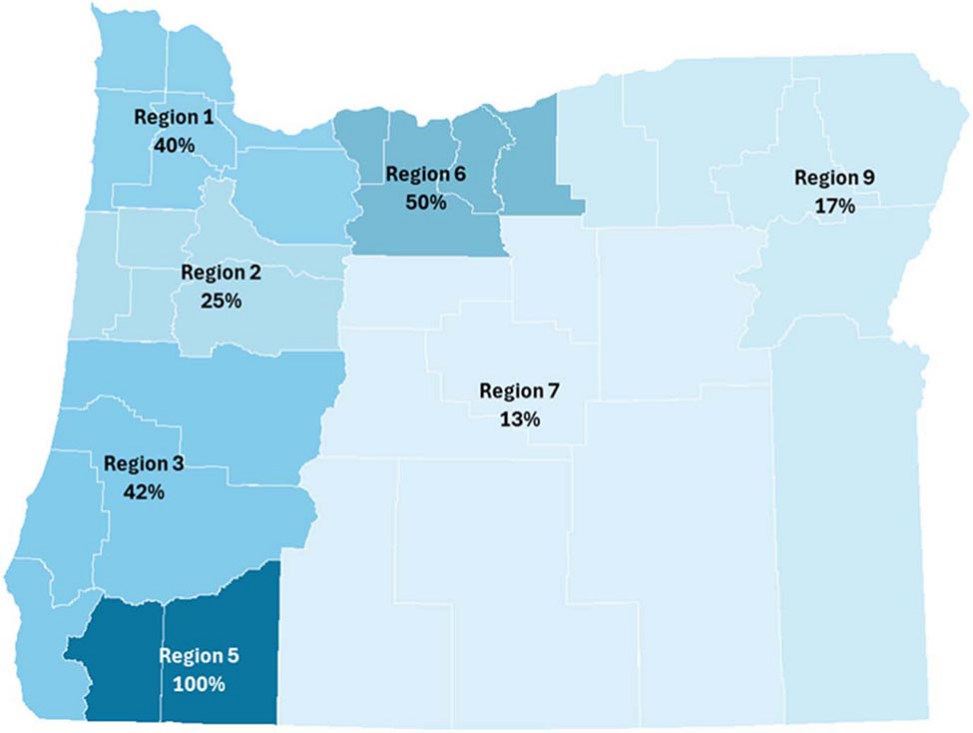
Percent of target healthcare facilities currently implementing routine admission screening (n=17) or actively working towards implementation (n=5) by geographic region

**Figure 4:**
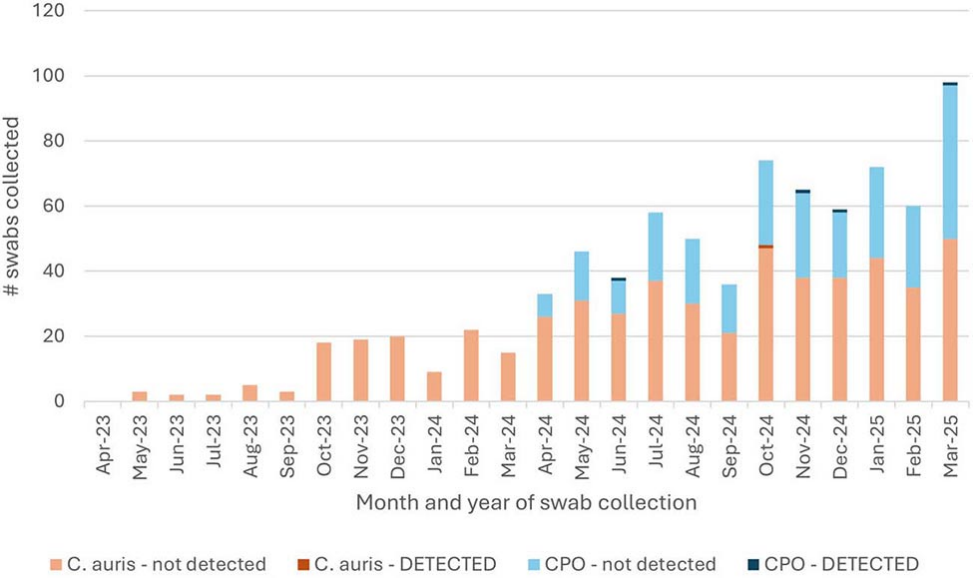
Monthly number of C. auris and CPO swabs collected over time* *OHA relies on healthcare facility partners to voluntarily report the number of swabs collected and tested at labs other than OSPHL and ARLN. Positive results require mandatory reporting.

**Results:**

An implementation timeline is shown in figure 2. As of 05/01/2025, 16 ACHs across six healthcare systems and one vSNF implemented routine admission screening; five additional ACHs are working toward it; and all other facilities are supported on an ad-hoc basis. At least one facility in each Oregon region is participating (figure 3). Screening criteria vary by facility or system. Facilities use a combination of labs to support testing including Oregon State Public Health Lab (7), Antibiotic Resistance Laboratory Network (5), private reference labs (4), and in-house clinical labs (1). When non-public health labs are used for screening, OHA relies on voluntary reporting of swab counts. Between 05/01/2023 and 03/31/2025, facilities collected 807 swabs; one of 543 *C. auris* swabs and four of 264 CPO swabs tested positive. Statewide screening has increased over time (figure 4).

**Conclusion:**

Admission screening for *C. auris* and CPOs can identify cases that would otherwise remain undetected in low-prevalence regions. Challenges include resource allocation, training clinical and laboratory staff, turnaround time, and data sharing when non-public health labs are used. Oregon’s experience can guide other low-prevalence jurisdictions initiating admission screening.

**Disclosures:**

Christopher D. Pfeiffer, MD, MHS, Department of Defense/MedPace: Grant/Research Support

